# Optimization of Extraction Process by Response Surface Methodology, Composition Analysis and Antioxidant Activity of Total Flavonoids from *Scutellaria baicalensis* Georgi

**DOI:** 10.3390/molecules31030507

**Published:** 2026-02-02

**Authors:** Huanwei Gan, Weiwei Lan, Min Wang, Jingyi Xu, Kaiyun Zhang, Ye Tang, Xin Gao, Aikedai Kadier, Chen Chen, Jianguo Wu, Tingting Liu, Ci Jin, Guilong Yan, Yuzhen Zhou

**Affiliations:** 1 Jiangsu Key Laboratory for Eco-Agricultural Biotechnology Around Hongze Lake, School of Life Science, Huaiyin Normal University, Changjiang West Road 111, Huai’an 223300, China; 2087721895@qq.com (H.G.); 471245895@qq.com (W.L.); 1420024671@qq.com (M.W.); 2293595601@qq.com (J.X.); zzzky1017@163.com (K.Z.); 428380165@qq.com (Y.T.); 2208593554@qq.com (X.G.); 3173919219@qq.com (A.K.); 1040113941@qq.com (C.C.); 709301295@qq.com (T.L.); 905587501@qq.com (C.J.); 56819817@qq.com (G.Y.); 99799890@qq.com (Y.Z.); 2 Collaborative Innovation Center Jointly Built by Province and Ministry of Regional Modern Agriculture and Environmental Protection, Changjiang West Road 111, Huai’an 223300, China

**Keywords:** *Scutellaria baicalensis* Georgi, flavonoid, response surface methodology, composition analysis, antioxidant activity

## Abstract

In order to further enhance the extraction yield of total flavonoids from *Scutellaria baicalensis* Georgi, the extraction process was optimized, and its composition and antioxidant activity were also analyzed. Through single-factor and response surface methodology optimization, the optimal extraction process of total flavonoids from *S. baicalensis* was obtained as follows: 56% ethanol concentration, 40:1 (mL/g) ratio of liquid to solid, 50 °C extraction temperature, and 1 h of extraction time. Under the optimal extraction conditions, the total flavonoid yield reached was 165.40 mg/g, which was 70.16% higher than the blank group and 89.68% higher than previously reported results. The major composition of total flavonoids was analyzed using UHPLC-MS/MS. A total of 60 flavonoid compounds were identified, of which 20 flavonoids had not been reported previously. The in vitro antioxidant activity of the total flavonoids was analyzed by DPPH and ABTs assays. IC_50_ of the total flavonoids on DPPH and ABTs free radicals were 0.52 μg/mL and 0.66 μg/mL, respectively, which indicated that the total flavonoids of *S. baicalensis* had a remarkable free radical scavenging ability. This study should provide theoretical and technical support for the industrial production and bioactivity-oriented utilization of flavonoids from *S. baicalensis*.

## 1. Introduction

*Scutellaria baicalensis* Georgi, a perennial herb in the Lamiaceae family, is widely used in Traditional Chinese Medicine, which is valued for both its culinary and therapeutic applications [1,2]. *S. baicalensis* possesses significant medicinal value, with its dried roots being the primary part used in medicine. It is characterized by a bitter taste and a cold nature, and is known for its diverse pharmacological effects, including clearing heat and dampness, purging fire, detoxification, stopping bleeding, and calming the fetus. Modern pharmacological studies have shown that *S. baicalensis* contains various bioactive compounds, such as flavonoids, alkaloids, polysaccharides, organic acids, trace elements, and volatile oils [3,4,5,6,7,8]. Flavonoids, such as baicalin, baicalein, wogonoside and wogonin in *S. baicalensis*, exhibit significant biological activities, including antioxidant, anti-inflammatory, antimicrobial, antitumor, and immunomodulatory effects, underpinning their substantial potential for applications across pharmaceutical, nutritional, and cosmetic fields [9,10]. Flavonoids demonstrate antioxidant effects by directly scavenging free radicals (including superoxide anions, hydroxyl radicals, and DPPH), chelating metal ions (thus inhibiting metal-catalyzed radical generation), and activating the Nrf2/ARE signaling pathway, ultimately maintaining cellular redox homeostasis to prevent and treat oxidative stress-related diseases [11,12]. Antioxidant activity is one of the most significant biological functions of flavonoids [13]. With the deepening of research on the bioactive components of natural products, the efficient extraction of flavonoids has become a critical step in developing related products [14].

The extraction methods of flavonoids from *S. baicalensis* mainly include solvent extraction, ultrasonic-assisted extraction, microwave-assisted extraction, enzymatic hydrolysis extraction, supercritical CO_2_ extraction, and ionic liquid + microwave/ultrasound combined extraction [15,16,17,18]. However, compared to the flavonoids extraction from other plant sources, current extraction processes for flavonoids of *S. baicalensis* have limitations in extraction yield, energy efficiency, and environmental impact [19]. The most common technique for isolating flavonoids from *S. baicalensis* was solvent extraction, including decoction, reflux extraction, and Soxhlet extraction. While these methods are straightforward to perform, they are hampered by several drawbacks, such as low extraction yields, substantial solvent use, and lengthy extraction durations [20]. Modern extraction techniques, including ultrasonic-assisted extraction, microwave-assisted extraction, supercritical fluid extraction, and enzymatic hydrolysis, have operational benefits such as improved efficiency, reduced solvent usage, and shortened processing times, but usually require higher equipment investment [21,22]. Response surface methodology (RSM) has been used to systematically optimize key parameters such as solvent concentration, extraction time, and ratio of liquid to solid to improve the extraction efficiency of flavonoids from *S. baicalensis*. Nevertheless, due to structural heterogeneity, chemical composition differences, and inconsistent optimization of extraction parameters, the extraction rates of flavonoids from different plant sources vary significantly. The application of RSM improved process performance and increased the yield of active compounds while reducing the number of variables through selective focus on critical factors, thereby decreasing operational costs and experimental time [23,24].

So far, the total flavonoid yield from *S. baicalensis* has been generally low. Due to different extraction techniques, the flavonoid compounds isolated and identified from *S. baicalensis* have also been limited. Therefore, in order to further improve the yield of the total flavonoids and complement the flavonoid compounds in *S. baicalensis*. In this study, the extraction process of total flavonoids was optimized using RSM, and then composition analysis and antioxidant activities were also studied. The results should provide theoretical and technical support for the industrial production and bioactivity-oriented utilization of flavonoids of *S. baicalensis*.

## 2. Results and Discussion

### 2.1. Single-Factor Optimization

**Figure 1:**
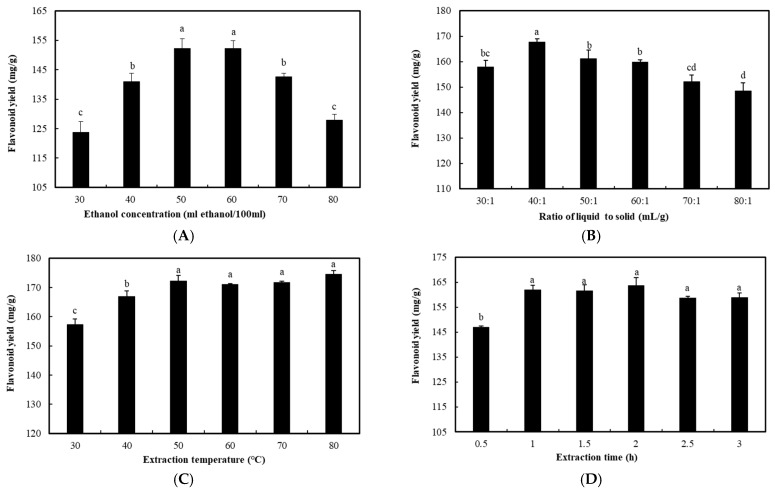
Effects of different single-factor extraction parameters on the extraction of flavonoids from *S. baicalensis*. (**A**): Ethanol concentration; (**B**): ratio of liquid to solid; (**C**): extraction temperature; (**D**): extraction time. Bars indicated standard errors (±SE). Different letters denoted significant differences (*p* < 0.05).

Ethanol concentration, ratio of liquid to solid, extraction temperature and extraction time were the most important factors affecting the extraction of flavonoids. The experimental results are shown in Figure 1.

Ethanol was widely used as a solvent for flavonoid extraction due to its broad solubility for various compounds [25]. The effect of ethanol concentration ranging from 30% to 80% on flavonoid extraction from *S. baicalensis* was studied. When ethanol concentration was 50% or 60%, the total flavonoid yield reached its maximum value of 152.33 mg/g, as shown in Figure 1A. When the ethanol concentration was too low, the flavonoids were not fully dissolved; when the ethanol concentration was too high, the alcohol-soluble substances, lipophilic components, and plant pigments in *S. baicalensis* were dissolved [26], competing with the flavonoids for dissolution, which reduced the efficiency of flavonoid extraction. As shown in Figure 1B, the ratio of liquid to solid had a significant impact on the extraction efficiency of total flavonoids. Generally, due to the limited solubility of flavonoids in solvents, the yield of the extract increased with the increase in the ratio of liquid to solid. Using a low solvent volume might provide a complete contact surface area with the plant material within a certain range, resulting in a gradual decrease in the total flavonoid extraction rate. Choosing an appropriate concentration was beneficial for the dissolution of flavonoids. The total flavonoid yield was increased when the ratio was raised from 30:1 to 40:1 (mL/g) but decreased with further increases in the ratio. As shown in Figure 1C, extraction temperature also has a significant impact on the extraction efficiency of total flavonoids. The flavonoid yield increased with the increase in extraction temperature. Elevated temperatures could improve extraction efficiency and accelerate the extraction process because higher temperatures increase the kinetic energy of molecules, leading to greater solubility and diffusion, which was conducive to the dissolution of flavonoids and the rupture of cell walls. The flavonoid yield increased with the temperature increases. After the temperature increased to 50 °C, the flavonoid yield remained stable. As shown in Figure 1D, the flavonoid yield first increased and then stabilized with the increase in extraction time. Prolonging extraction time might improve extraction efficiency, but there is a risk of oxidative degradation of active compounds [27], while extending heating could cause flavonoid decomposition. Therefore, the optimal single-factor conditions of flavonoid extraction from *S. baicalensis* were 50–60% ethanol concentration, 40:1 ratio of liquid to solid, 50 °C extraction temperature, and 60 min of extraction time. Based on these optimal values as the center point, the response surface methodology was used to further optimize the extraction parameters.

### 2.2. Optimization by Response Surface Methodology

Ethanol concentration, ratio of liquid to solid, and extraction time significantly influenced the total flavonoid yield, and the Box-Behnken design (BBD) in RSM was implemented to determine the optimal levels of the three selected factors (*A* for ethanol concentration, *B* for ratio of liquid to solid, and *C* for extraction time). The experimental design and results are shown in Table 1.

Multiple regression coefficients were calculated using the least squares method, and a quadratic polynomial model was developed to predict the measured response variables. The following regression equation was obtained by application of RSM as shown in Equation (1).

(1) $$Y=-341.07+6.60A+9.12B+4.05C-0.065A^{2}+0.0053AB+0.0062AC-0.124B^{2}+0.0091BC-0.036C^{2}$$

The analysis of variance data (ANOVA) for the selected quadratic polynomial model is listed in Table 2. The model demonstrated high significance with an *F*-value of 9.54 and *p* < 0.05, which indicated the model’s adequacy for predicting experimental outcomes [28,29]. The coefficients of determination *R*^2^ and adjusted *R*^2^ were calculated as 0.945 and 0.8459, respectively, which implied that the model was reliable for the total flavonoid yield. Among the model terms, linear term *A* (ethanol concentration) and *C* (extraction time) and the quadratic terms *A*^2^ and *C*^2^ were statistically significant (*p* < 0.05). In contrast, linear term *B* (ratio of liquid to solid), interaction terms (*AB*, *AC*, *BC*), and quadratic term *B*^2^ showed no significant effects on the total flavonoid yield (*p* > 0.05). The relative influence of factors on extraction efficiency followed this order: *A* > *C* > *B* as determined by *F*-values. This quantitative assessment confirmed that ethanol concentration was the most significant factor, while ratio of liquid to solid showed the least influence.

**Figure 2:**
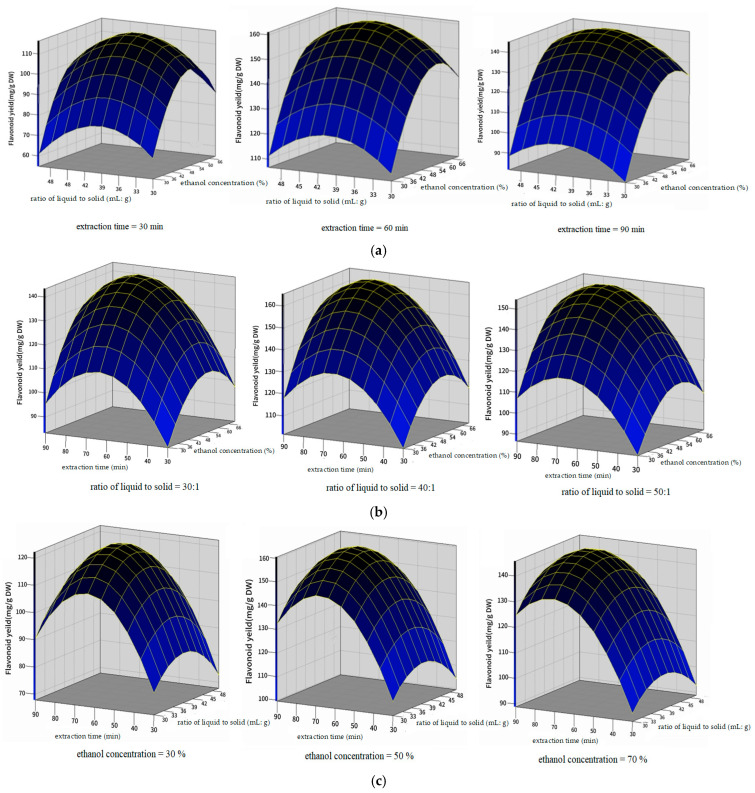
The 3D response surfaces of interaction of various factors on the total flavonoid yield. (**a**): ratio of liquid to solid and ethanol concentration; (**b**): extraction time and ethanol concentration; (**c**): extraction time and ratio of liquid to solid.

The 3D response surfaces (Figure 2) generated by SAS 9.4 are the graphical representations of Equation (1). They visualize the relationship between the response and each variable as well as the interactions between two tested variables. As shown in Figure 2a, when the extraction time was 30 min, 60 min, and 90 min, the total flavonoid yield was in the range from 61.2 to 112.1 mg/g, 109.4 to 156.3 mg/g, and 82.3 to 142.5 mg/g, respectively. When the extraction time remained constant, the total flavonoid yield could reach its maximum value when the ethanol concentration and ratio of liquid to solid were close to their optimal values. As shown in Figure 2b, when the ratio of liquid to solid was 30:1, 40:1, and 50:1, the total flavonoid yield was in the range from 84.5 to 140.1 mg/g, 102.6 to 161.3 mg/g, and 87.8 to 151.4 mg/g, respectively. When the ratio of liquid to solid remained constant, the total flavonoid yield first increased and then decreased with the increase in the ratio of liquid to solid. As shown in Figure 2c, when the ethanol concentration was 30%, 50%, and 70%, the total flavonoid yield was in the range from 73.5 to 116.7 mg/g, 105.6 to 155.9 mg/g, and 92.4 to 141.4 mg/g, respectively. When the ethanol concentration remained constant, the optimization of extraction time and ratio of liquid to solid was beneficial for the improvement of the total flavonoid yield. In conclusion, all response surfaces exhibited downward-opening parabolic shapes, which confirmed the existence of maximum values for the total flavonoid yield. Each response surface showed a distinct peak, which demonstrated that the total flavonoid yield initially increased and then decreased with rising ethanol concentration, ratio of liquid to solid, and extraction time.

The optimum conditions of ethanol concentration, ratio of liquid to solid, and extraction time could be determined by ridge analysis, as shown in Table 3. On the basis of the ridge analysis, it was predicted that the maximum flavonoids yield of 163.13 mg/g could be reached when ethanol concentration, ratio of liquid to solid, and extraction time were 56.30%, 40.32:1 (mL/g), and 67.34 min, respectively. However, considering practical operational feasibility and resource efficiency, the parameters were adjusted to 56% ethanol concentration, 40:1 mL/g ratio of liquid to solid, and 60 min of extraction time, and the predicted flavonoids yield was 160.20 mg/g according to Equation (1). To verify the predicted results, three validation experiments were conducted. Under the optimized condition, the total flavonoid yield reached 165.40 mg/g (*SD* = 0.23%), which implied that experimental and predicted values (160.20 mg/g) were in good agreement.

Xiang et al. [30] used ultrasonic-microwave-assisted extraction to extract flavonoids from *S. baicalensis* by the response surface methodology. Under the optimal process, the total flavonoid yield was only 87.1 mg/g, which is shown in Table 4. In this study, the total flavonoid yield increased to 89.67%. Analyzing the differences in extraction processes between the two, it was found that there were significant differences between this study and the literature in terms of ratio of liquid to solid and extraction time. The optimal ratio of liquid to solid obtained was only 24:1, and the extraction time was only 15 min in the literature. Therefore, a lower ratio of liquid to solid and a shorter extraction time could not effectively extract all flavonoids from *S. baicalensis*. In addition, Lim et al. [12] and Liu et al. [23] both used the ethanol method to extract flavonoids from *S. baicalensis*, but their extraction yields (40.11 mg/g and 19.437 mg/g, respectively) were much lower than the results of this study because of differences in optimization parameters.

### 2.3. Chemical Composition Analysis

**Figure 3:**
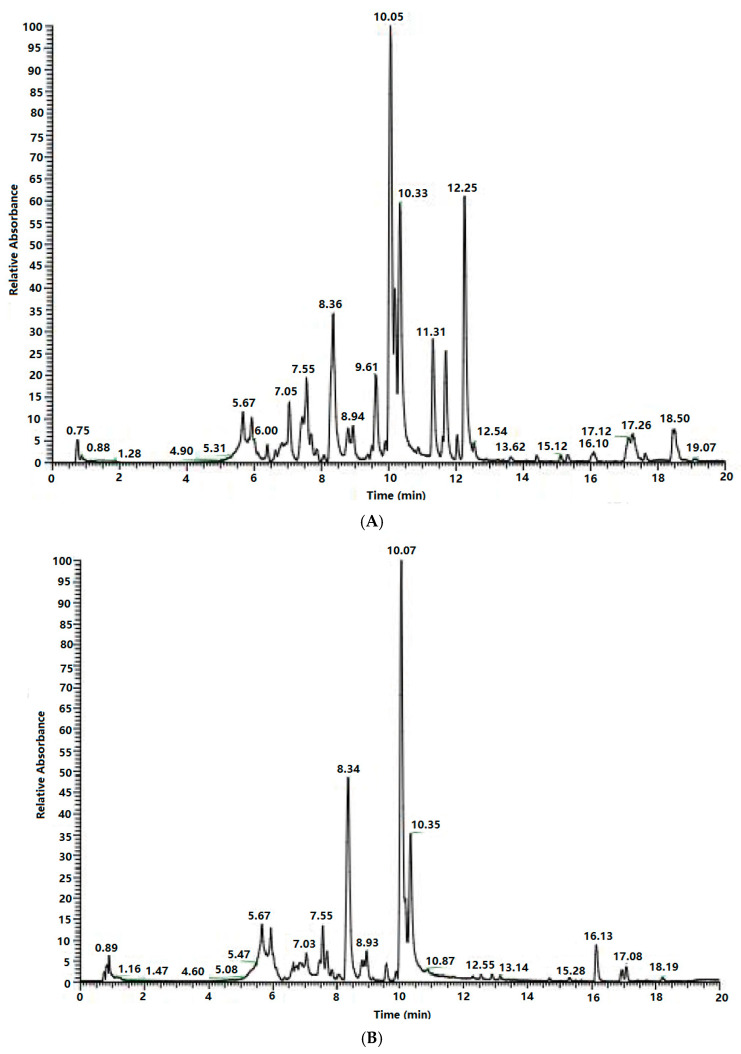
Total ion chromatogram of the total flavonoid extract. (**A**): Positive ion pattern; (**B**): negative ion pattern.

The phytochemical composition of the extract was characterized using UHPLC and analyzed through data-dependent acquisition (DDA) in both positive and negative ion modes, which are shown in Figure 3. The LC-MS data were processed using the Compound Discoverer software. Sixty types of flavonoids with high matching scores were identified as shown in Table 5, of which 20 flavonoids had not been reported previously. The flavonoids were identified and classified into five major categories, including flavones, isoflavones, flavonols, dihydroflavones, and chalcones. It was worth noting that twenty flavonoids lacked sufficient literature records, which indicated that they might be novel or rarely reported compounds in *S. baicalensis*.

Thirty-eight flavones had been identified, such as wogonin, oroxylin A, tectochrysin, baicalin and baicalein. These compounds had been shown to possess significant antioxidant, anti-inflammatory, anti-tumor and neuroprotective properties [31,43]. However, it should be noted that these flavones exhibited limited water solubility and absorption. Due to the glycosyl modification, the water solubility of flavone glycosides (for example, baicalin and wogonin) were enhanced. The release of aglycones following intestinal hydrolysis was necessary to enhance bioavailability, which was deemed suitable for liver protection, hypoglycemia and cardiovascular protection [52]. It has been demonstrated that both of them possess the capacity to resist bacterial infection and regulate immunity [53]. However, aglycones have been shown to demonstrate rapid and direct action, while more stable glycosides are more suitable for oral administration. The total flavones extracted contained various flavonoid derivatives because of hydroxylation and methoxylation, such as dihydroxy dimethoxyflavoid, trihydroxy dimethoxyflavoid, and trihydroxy trimethoxyflavoid. Lysionotin is a naturally occurring flavone mainly extracted from the plant *Lysionotus pauciflorus* Maxim. To date, there has been a lack of literature reports that clearly confirm the presence of lysonotin in *S. baicalensis*. 6-demethoxytangeretin is a trimethoxyflavone commonly found in citrus plants. Clearly, 6-demethoxytangeretin was not the main component of *S. baicalensis*. However, the possibility of the presence of trace amounts of 6-demethoxytangeretin in *S. baicalensis* could not be ruled out. The chemical composition of plants is influenced by various factors, and due to differences in geographical location, growth conditions, or extraction processes, it could not be completely excluded that *S. baicalensis* may contain trace amounts of lysionotin and 6-demethoxytangeretin. Therefore, future research should focus on the content of *S. baicalensis* from different sources to further clarify its chemical composition.

Ten isoflavones were identified, including 5-O-methylgenistein, puerarin and tectoridin, amongst others. These compounds, found in plants, have been proven to possess estrogenic activity. The ability of these substances to regulate endocrine function in both directions has been demonstrated, as has their effectiveness in alleviating menopausal symptoms and preventing osteoporosis [54,55]. They protect the cardiovascular system through antioxidant and anti-inflammatory effects, reduce cholesterol, and improve arteriosclerosis. Isoflavones could also inhibit the proliferation of tumor cells, especially having a preventive effect on hormone-dependent cancers (such as breast cancer and prostate cancer) and possessing antibacterial and antiviral properties that help enhance immunity [56,57]. Currently, sophoricoside, irigenin, iridin, isotectorigenin-7-methyl ether, dipteryxin, and iristectorigenin B have not been reported in *S. baicalensis*.

There were six types of flavonols which can scavenge free radicals, inhibit lipid peroxidation, and protect cells from oxidative damage. Quercetin has anti-inflammatory effects achieved through regulating pathways such as NF-κB and COX-2, which could alleviate chronic inflammation [58,59]. Flavonols can also enhance vascular elasticity, lower blood pressure, prevent cardiovascular diseases, and inhibit tumor cell proliferation and metastasis [60]. Eupafolin is an O-methylated flavonoid commonly found in plants of the *Asteraceae* family. It has exhibited various biological activities such as antioxidant and anti-inflammatory properties. Kaempferol-7-O-glucoside is a glycoside form of kaempferol. The types and contents of flavonoids vary significantly among different plants. Currently, there is no direct evidence indicating the presence of fisetin, eupafolin, quercitrin or kaempferol-7-O-glucoside in *S. baicalensis*. However, as an important medicinal plant, its main active ingredients, such as flavonoids like baicalin, baicalein, and wogonoside, have been extensively studied and proven to possess various pharmacological activities. Nonetheless, trace amounts of fisetin, eupafolin, quercitrin and kaempferol-7-O-glucoside might exist.

There were four types of dihydroflavonoids found, including taxifolin, alpinetin, and pinocembrin, which are flavonoid derivatives with a C2–C3 single bond structure. They exhibit significant antioxidant, anti-inflammatory, and neuroprotective effects, capable of alleviating oxidative stress and neurodegenerative damage [61]. Dihydroflavonoids inhibit inflammatory responses by regulating the NF-κB and MAPK pathways and improve cardiovascular function, such as reducing blood lipids and enhancing vascular elasticity [62,63]. Dihydroflavonoids also exhibit anti-tumor activity, are capable of inducing apoptosis in cancer cells, and possess both antidepressant and anxiolytic effects [64]. Gerberinside is a newly discovered dihydroflavonoid derived from *S. baicalensis*.

There were two types of chalcones found, including cardamonin and flavokawain A. Chalcones exhibit significant antioxidant and anti-inflammatory effects, effectively scavenging free radicals and inhibiting the release of inflammatory factors. Chalcones also possessed remarkable anti-tumor activity, exerting their anticancer effects by regulating the cell cycle, inducing apoptosis, and inhibiting angiogenesis. Furthermore, chalcones have exhibited multiple functions such as antibacterial, antiviral, and neuroprotective effects and regulating glucose and lipid metabolism [65,66,67]. The skeleton of chalcone has “chemical plasticity” and “biological controllability” and has become a truly efficient lead compound platform [68].

Using a high-resolution liquid chromatography-mass spectrometry instrument, various characteristic flavonoids such as baicalin, baicalein, wogonoside, and wogonin were identified, which are known as the characteristic flavonoid components of *S. baicalensis*. Additionally, some other compounds were detected, but there was a lack of literature support. Due to the influence of various factors on plant chemical composition, it could not be completely ruled out that all components of *S. baicalensis* might not be detected due to differences in geographical location, growth conditions, or extraction processes. These might include trace amounts of lysionotin, 6-demethoxytangeretin, eupafolin, cardamonin, and other flavonoids. Therefore, future research could focus on the content of *S. baicalensis* from different sources, derivatives produced during extraction, trace secondary metabolites, or impurities and should be combined with nuclear magnetic resonance (NMR) data, standard sample comparison, and more literature evidence to further clarify its chemical composition.

### 2.4. Antioxidant Activity

The spectrophotometric method is a widely employed technique for the evaluation of antioxidant performance, offering particular value in the acquisition of information regarding the inherent redox activity of plant extracts due to their capacity to promote health [69]. This study evaluated the decolorizing effects of flavonoids from *S. baicalensis* on synthetic free radicals, non-physiological free radicals, and colored free radicals using the DPPH and ABT_S_ methods, with vitamin C (V_C_) as a control. These free radicals could be scavenged by redox-active compounds through one or two electron transfers [70]. The rapid change in absorbance indicated that the flavonoid extract exhibited stronger antioxidant activity in terms of its ability to provide hydrogen atoms.

**Figure 4:**
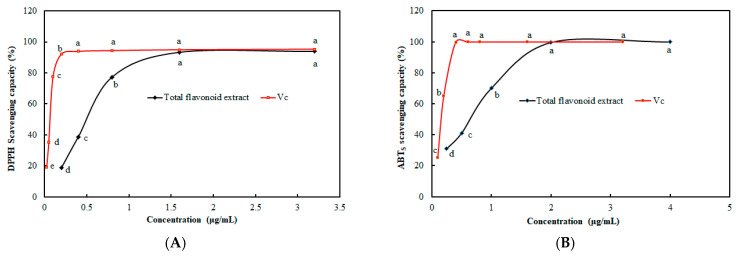
Analysis of antioxidant activity of total flavonoid extract. (**A**): DPPH radical scavenging capacity; (**B**): ABT_S_ radical scavenging capacity. Groups with distinct letters were significantly different at *p* < 0.05.

As showed in Figure 4A, the scavenging ability of the total flavonoids on DPPH free radicals increased with the increase in the total flavonoid concentration within the concentration range of 0–1.6 μg/mL. When the concentration of the total flavonoids exceeded 1.6 μg/mL, the DPPH free radical scavenging rate stabilized at 96.60%, which approached that of V_C_. The IC_50_ of the total flavonoids on DPPH free radicals was only 0.52 μg/mL. As shown in Figure 4B, the scavenging ability of the total flavonoids on ABT_S_ increased with the increase in the total flavonoid concentration within the concentration range of 0.25~2 μg/mL. When the concentration was higher than 2 μg/mL, the ABT_S_ free radical scavenging rate tended to stabilize at 99.88%, which approached that of V_C_. The IC_50_ of the total flavonoids on ABT_S_ free radicals were 0.66 μg/mL. Lim et al. [12] extracted the total flavonoids from *S. baicalensis* using 70% aqueous ethanol, and IC_50_ of the total flavonoids on DPPH and ABTs free radicals were 0.49 mg/mL and 0.29 mg/mL, respectively, which were all higher than the values of this study. Accordingly, the total flavonoids from *S. baicalensis* in this study exhibited excellent scavenging ability against DPPH and ABT_S_ free radicals, which indicated that the total flavonoids had an excellent antioxidant capacity.

## 3. Materials and Methods

### 3.1. Materials and Agents

Roots of *S. baicalensis* were purchased from Beijing Tongrentang Pharmacy, Beijing China. Baicalein (98% purity) was purchased from Shanghai Yuanye Biotechnology Co., Ltd, Shanghai, China. Vc was sourced from Huazhong Pharmaceutical Co., Ltd, Xiangyang, China. 1,1-diphenyl-2-picrylhydrazyl (DPPH) and 2,2′-azino-bis (3-ethylbenzothiazoline-6-sulfonic acid) (ABT_S_) were purchased from Shanghai Jieshikang Biotechnology Co., Ltd., Shanghai, China. Ethanol, sodium nitrite, anhydrous aluminum chloride, and sodium hydroxide were all analytical grade and purchased from Sinopharm Chemical Reagent Co., Ltd, Shanghai, China.

### 3.2. Initial Extraction Methods

Roots of *S. baicalensis* were dried to a constant weight, crushed by a universal pulverizer (DE-300g, Zhejiang Hongjingtian Industrial & Trading Co., Ltd, Jinhua, China), and then sieved through a 40-mesh sieve. A 0.5 g of *S. baicalensis* powder was mixed with 20 mL of 60% ethanol (*v*/*v*), which was placed in a water bath (SHZ-B, Changzhou Nuoji Instrument Co., Ltd, Changzhou, China) at 50 °C for 1 hour. Then, the mixture was centrifuged at 5000 r/m for 10 min in a high-speed large-capacity centrifuge (H2050, Shanghai Luxiangyi Centrifuge Instrument Co., Ltd, Shanghai, China). The supernatant of the total flavonoid extract was collected for determination [71].

### 3.3. Single-Factor Extraction Methods

Based on the initial extraction conditions, the effects of single factors on the extraction of flavonoid from *S. baicalensis* were investigated by varying the ethanol concentration, ratio of liquid to solid, extraction temperature, and extraction time individually. Specifically, the ethanol concentration changed from 30% to 80% (*v*/*v*) under the conditions of 40:1 liquid to solid ratio at 50 °C for 1 h; ratio of liquid to solid changed from 30:1 to 70:1 under the conditions of 60% ethanol (*v*/*v*) and 50 °C for 1 h; extraction temperature changed from 30 °C to 80 °C under the conditions of 60% ethanol (*v*/*v*) and 40:1 liquid to solid ratio for 1 h; and extraction time changed from 0.5 h to 3 h under the conditions of 60% ethanol (*v*/*v*) and 40:1 liquid to solid ratio at 50 °C.

### 3.4. Response Surface Methodology Design

Based on the results of single-factor extraction, a Box-Behnken design was implemented to evaluate the effects of three independent variables including ethanol concentration, ratio of liquid to solid, and extraction time on the total flavonoid yield [72]. All experiments were conducted according to the design matrix shown in Table 1. Regression analysis was performed using the statistical software package Synthetically Awesome Stylesheets 9.4 to estimate coefficients of the regression equation. Multiple regression analysis was conducted to establish an empirical model between the measured response and independent variables. The second-order response function (Equation (2)) was predicted by the following equation:

*Y* = a_0_ + a_1_*A* + a_2_*B* + a_3_*C* + a_11_*A*^2^ + a_12_*AB* + a_13_*AC* + a_22_*B*^2^ + a_23_*BC* + a_33_*C*^2^ (2)

where *A*, *B*, and *C* represent the three independent variables, namely, ethanol concentration, ratio of liquid to solid, and extraction time, respectively; a_0_, a_1_, a_2_, a_3_, a_11_, a_12_, a_13_, a_22_, a_23_, and a_33_ were the coefficients of independent variables.

### 3.5. Determination of Total Flavonoids

Due to the presence of numerous flavonoid compounds in *S. baicalensis*, it was hard to accurately determine the total flavonoid concentration using UV spectrophotometry. Therefore, UV spectrophotometry following aluminum chloride chromogenic reaction was used to determine the concentration of the total flavonoids [73]. Specifically, 1 mL of diluted flavonoid extract was mixed with 0.3 mL of 5% NaNO_2_ solution and allowed to stand for 6 min. Then, 0.3 mL of 10% AlCl_3_ solution was added and allowed to stand for 6 min. Subsequently, 4 mL of 1 M NaOH solution was added, and the volume was adjusted to 15 mL with distilled water. After shaking the mixture well, the mixture stood for 20 min at room temperature. The absorbance was measured at 378 nm using an UV spectrophotometer (EU-2800R, Shanghai Angla Instrument Co., Ltd., Shanghai, China). The concentration of total flavonoids was determined by substituting the absorbance value into the regression equation derived from the standard curve of baicalein. The flavonoid yield was calculated using Equation (3).

(3) $$Y=\frac{C\times N\times V}{M}\times 100\%$$

where Y is the flavonoid yield, mg/g; C is the concentration of the total flavonoids, mg/mL; V is the total volume of extract, mL; N is the dilution factor; and M is the mass of *S. baicalensis* powder, g.

### 3.6. UPLC-Q-Exactive HESI-MSH-MS Analysis

The analysis of flavonoid components in the total flavonoid extract was conducted using the UPLC-Q-Exactive HESI-MSH-MS method.

The chromatographic separation was performed on a Thermo Scientific Ultimate 3000 UHPLC system (Thermo Fisher Scientific, Carlsbad, CA, USA) equipped with a C18 column (1.9 µm, 2.1 mm × 100 mm). The analysis was conducted with a 0.3 mL/min flow rate and 10 μL of injection volume. The mobile phase consisted of 0.1% formic acid/acetonitrile (B) and 0.1% formic acid/water (A). The gradient elution program was as follows: 0 min, 10% of mobile phase B; 10 min, 100% of mobile phase B; 15 min, 100% of mobile phase B; 17.1 min, 10% of mobile phase B; 20 min, 10% of mobile phase B.

Mass spectrometric analysis was conducted on a Q-Exactive mass spectrometer (Thermo Fisher Scientific, Carlsbad, CA, USA) with a HESI ion source. The source parameters were set as follows: 310 °C of ion source temperature, 320 °C of capillary temperature, 30 units of sheath gas flow rate, 10 units of auxiliary gas flow rate, 3 kV of spray voltage in positive ion mode, and 2.8 kV of spray voltage in negative ion mode. Data-dependent analysis (DDA) was used for analysis, with loop count set to 10. HCD energy adopted step-wise normalized collision energy, with set values of 10, 28, and 35 eV. The first-order mass spectrum scanning range was 100–1500 *m*/*z*, with a resolution of 70,000, and the AGC target was set to 3 × 10^6^ with 200 ms of injection time. The second-order mass spectrum resolution was set to 17,500, and the AGC target was set to 1 × 10^5^ with 50 ms of injection time.

The LC-MS data were processed using Compound Discoverer software (V3.2, Thermo Fisher Scientific, Carlsbad, CA, USA) for automated compound identification against multiple databases, including Chemspider Plant Database, CHEBI, CHEMBL, Natural Products Database, Flavonoids Database, OTC Traditional Chinese Medicine Database, and mz Cloud Database.

### 3.7. Analysis of Antioxidant Activity

#### 3.7.1. DPPH Radical Scavenging Activity

DPPH radical scavenging activities of the total flavonoid extract from *S. baicalensis* were conducted as follows: 2.0 mL of the appropriately diluted total flavonoid extract was mixed with 2.0 mL of 1.37 × 10^−4^ mg/mL DPPH solution, and then the mixture was kept in darkness for 30 min for measurement at 517 nm [74]. The DPPH radical scavenging rate was calculated using Equation (4). V_C_ was used as a positive control and processed under identical conditions for comparative analysis.

(4) $$\mathrm{DPPH}\;\mathrm{radical}\;\mathrm{scavenging}\;\mathrm{rate}=\left(1-\frac{A_{1}-A_{2}}{A_{3}}\right)\times 100\%$$

where A_1_ is the absorbance of the sample mixed with the DPPH reagent; A_2_ is the absorbance of the sample mixed with anhydrous ethanol; and A_3_ is the absorbance of DPPH reagent mixed with anhydrous ethanol.

#### 3.7.2. ABT_S_ Radical Scavenging Activity

The ABT_S_ radical scavenging activity of the total flavonoid extract from *S. baicalensis* was conducted as follows: 0.8 mL of the appropriately diluted total flavonoid extract was mixed with 3.2 mL of ABT_S_ solution, and then the mixture was kept in darkness for 30 min for measurement at 734 nm [75]. The ABT_S_ radical scavenging rate was calculated using Equation (5). V_C_ was used as a positive control and processed under identical conditions for comparative analysis.

(5) $${\mathrm{ABT}}_{S}\;\mathrm{radical}\;\mathrm{scavenging}\;\mathrm{rate}=\left(1-\frac{A_{1}-A_{2}}{A_{3}}\right)\times 100\%$$

where A_1_ is the absorbance of the sample mixed with the ABT_S_ reagent; A_2_ is the absorbance of the sample mixed with anhydrous ethanol; and A_3_ is the absorbance of the ABT_S_ reagent mixed with anhydrous ethanol.

### 3.8. Statistical Analysis

Statistical analysis was performed using Microsoft Excel 2016 (Microsoft, Redmond, WA, USA) for data processing and SPSS 16.0 (IBM Corporation, Armonk, NY, USA) for statistical computations. Response surface methodology design and analysis, including coefficients of mathematical model, significance level, lack of fit, and adjusted R^2^, were conducted using Synthetically Awesome Stylesheets 9.4 (SAS Institute Inc., Cary, NC, USA).

## 4. Conclusions

In order to further improve the extraction yield of total flavonoids from *S. baicalensis*, single-factor and response surface methodology optimization were conducted, and the optimal extraction process of total flavonoids from *S. baicalensis* was obtained as followed: 56% ethanol concentration, 40:1 mL/g ratio of liquid to solid, 50 °C extraction temperature, and 1 h of extraction time. Under the optimal extraction conditions, the total flavonoid yield reached was 165.40 mg/g, which was 70.16% higher than the blank group and 89.68% higher than previously reported results. The major composition of the total flavonoids was analyzed using UPLC-MS. A total of 60 flavonoid compounds were identified, of which 20 flavonoids had not been reported previously. The in-vitro antioxidant activity of the total flavonoids was analyzed by DPPH and ABTs assays. IC_50_ of the total flavonoids on DPPH and ABT_S_ free radicals were 0.52 μg/mL and 0.66 μg/mL, respectively.

## Figures and Tables

**Table 1:** Design and results of response surface methodology.

No.	*A* Ethanol Concentration (%)	*B* Ratio of Liquid to Solid (mL/g)	*C* Extraction Time (min)	*Y* Total Flavonoid Yield (mg/g)
1	30	30:1	60	97.19
2	30	50:1	60	101.85
3	70	30:1	60	138.29
4	70	50:1	60	147.11
5	50	30:1	30	106.10
6	50	30:1	90	131.62
7	50	50:1	30	92.79
8	50	50:1	90	129.18
9	30	40:1	30	85.71
10	70	40:1	30	93.23
11	30	40:1	90	102.52
12	70	40:1	90	124.80
13	50	40:1	60	162.11
14	50	40:1	60	156.37
15	50	40:1	60	159.65

**Table 2:** Analysis of variance data on the total flavonoid yield.

Source	DF	Sum of Squares	Mean Square	*F*	*Pr* (>*F*)
*A*	1	1687.81	1687.81	15.47	0.011 *
*B*	1	0.66	0.66	0.0061	0.941
*C*	1	1520.76	1520.76	13.94	0.0135 *
*A* ^2^	1	2461.72	2461.72	22.57	0.0051 **
*AB*	1	4.41	4.41	0.040	0.849
*AC*	1	54.76	54.76	0.50	0.510
*B* ^2^	1	571.93	571.93	5.24	0.0707
*BC*	1	29.70	29.70	0.27	0.624
*C* ^2^	1	3779.94	3779.94	34.65	0.0020 **
Model	9	9363.01	1040.33	9.54	0.0115 *
Error	5	545.40	109.08	/	/
(Lack of fit)	3	529.08	176.36	21.60	0.045 *
(Pure error)	2	16.32	8.16	/	
Total	14	9908.41	/	/	/
R^2^ = 0.9450; Adj. R^2^ = 0.8459; coefficient of variation = 8.57					

* Indicates significance (*p* < 0.05); ** indicates extreme significance (*p* < 0.01).

**Table 3:** Ridge analysis for the optimum conditions of the total flavonoid yield.

Radius	*A* Ethanol Concentration (%)	*B* Liquid-Solid Ratio (mL/g)	*C* Extraction Time (min)	*Y* Predicted Response (mg/g)
0	50.00	40.00:1	60.00	159.37
0.1	51.49	40.00:1	62.00	161.10
0.2	53.05	40.03:1	63.89	162.30
0.3	54.66	40.12:1	65.66	162.98
0.4	56.30	40.32:1	67.34	163.13
0.5	57.91	40.72:1	68.91	162.78
0.6	59.35	41.54:1	70.29	161.95
0.7	60.36	42.82:1	71.32	160.70
0.8	60.96	44.23:1	72.02	159.13
0.9	61.36	45.60:1	72.53	157.26
1	61.65	46.89:1	72.95	155.12

**Table 4:** Comparison of extraction yield of total flavonoids from *S. baicalensis* in the major literature.

No.	Method	Extraction Yield (mg/g)	Year	Literature
1	70% aqueous ethanol	40.11	2024	[12]
2	Ethanol	19.437	2015	[23]
3	Ultrasonic-microwave-sassisted extraction	87.1	2017	[30]
4	Ethanol	165.40	2026	This study

**Table 5:** Sixty-one flavonoids of the extract identified by HESI-MSH-MS.

Classification	Name	Retention Time (min)	MW	Pattern *	Literatures	Name	Retention Time (min)	Mass	Pattern*	Literatures
Flavones	wogonin	10.055	284.07	PN	[31]	pectolinarigenin	7.394	314.08	N	[32]
	oroxylin A	10.337	284.07	PN	[31]	apigenin-7-*O*-glucuronide	7.497	446.08	PN	[33]
	tectochrysin	12.258	268.07	P	[34]	3′,4′,5,7-tetrahydroxyflavone	7.026	286.05	PN	[35]
	skullcapflavone II	10.182	374.10	PN	[36]	wogon-5-*O*-glucoside	7.374	446.12	P	[37]
	5,7,2-trihydroxy-8-methoxyflavone	8.348	300.06	PN	[38]	diosmetin	10.261	300.06	PN	[39]
	wogonoside	7.561	460.10	P	[31]	cirsimaritin	9.756	314.08	P	[40]
	5-hydroxy-6,7-dimethoxyflavone	11.318	298.08	P	[38]	jaceosidin	8.478	330.07	N	[41]
	5-demethylnobiletin	11.696	388.12	P	[42]	baicalein-7-*O*-glucuronide ethylester	8.402	474.12	N	[43]
	chrysin 6-*C*-glucoside 8-*C*-arabinoside	5.666	548.15	N	[33]	scutellarin	7.422	462.08	N	[31]
	baicalin	7.046	446.08	PN	[31]	oroxin A	7.479	432.11	P	[43]
	chrysin 6-*C*-arabinoside 8-*C*-glucoside	5.927	548.15	N	[33]	chrysin 7-glucuronide	6.399	430.09	P	[44]
	acacetin	7.489	284.07	N	[45]	apigenin 6,8-digalactoside	4.672	594.16	P	[44]
	chrysin	10.184	254.06	PN	[31]	dihydroxy dimethoxyflavonoid 1	10.116	314.08	PN	No
	gardenin B	12.038	358.11	P	[46]	trihydroxy dimethoxyflavonoid	8.793	330.07	PN	No
	baicalein	8.862	270.05	P	[31]	dihydroxy dimethoxyflavonoid 2	10.293	314.08	N	No
	6-*O*-methylbaicalin	7.697	476.10	N	[41]	trihydroxy trimethoxyflavonoid 1	9.905	344.09	PN	No
	oroxylin A-7-*O*-β-D-glucuronide	7.870	460.10	PN	[47]	trihydroxy trimethoxyflavonoid 2	10.533	344.09	P	No
	apigenin	8.420	270.05	PN	[35]	lysionotin	10.305	344.09	P	No
	norwogonin	8.864	270.05	PN	[48]	6-demethoxytangeretin	9.604	342.11	P	No
Isoflavones	5-*O*-methylgenistein	11.473	284.07	P	[49]	dipteryxin	10.131	314.08	P	No
	puerarin	6.374	416.11	P	[32]	iristectorigenin B	8.336	330.07	P	No
	tectoridin	6.940	462.12	PN	[50]	sophoricoside	7.823	432.11	PN	No
	7-*O*-β-glucopyranosyl-4′-hydroxy-5-methoxyisoflavone	6.288	446.12	P	[44]	irigenin	8.671	360.08	P	No
	isotectorigenin,7-methyl ether	11.778	328.09	P	No	iridin	6.601	522.14	PN	No
Flavonols	quercetin	6.120	302.04	N	[46]	eupafolin	7.646	316.06	PN	No
	kaempferol 3-glucuronide	5.913	462.08	PN	[51]	kaempferol 7-*O*-glucoside	5.214	448.10	N	No
	fisetin	8.499	286.05	PN	No	quercitrin	4.233	464.10	N	No
Dihydroflavones	taxifolin	5.384	304.06	P	[41]	pinocembrin	10.384	256.07	P	[46]
	alpinetin	8.730	270.09	P	[44]	gerberinside	6.971	338.10	PN	No
Chalcones	cardamonin	12.177	270.09	N	No	flavokawain A	13.187	314.12	N	No

*: P: positive ion pattern; N: negative ion pattern.

## Data Availability

The main data generated or analyzed to support the conclusion during this study are included in this published article. The full datasets generated and/or analyzed during the current study are available from the corresponding author by request.
